# Identification of diagnostic subnetwork markers for cancer in human protein-protein interaction network

**DOI:** 10.1186/1471-2105-11-S6-S8

**Published:** 2010-10-07

**Authors:** Junjie Su, Byung-Jun Yoon, Edward R Dougherty

**Affiliations:** 1 Department of Electrical and Computer Engineering, Texas A&M University, College Station, TX 77843-3128, USA; 2 Computational Biology Division, Translational Genomics Research Institute, Phoenix, AZ 85004, USA

## Abstract

**Background:**

Finding reliable gene markers for accurate disease classification is very challenging due to a number of reasons, including the small sample size of typical clinical data, high noise in gene expression measurements, and the heterogeneity across patients. In fact, gene markers identified in independent studies often do not coincide with each other, suggesting that many of the predicted markers may have no biological significance and may be simply artifacts of the analyzed dataset. To find more reliable and reproducible diagnostic markers, several studies proposed to analyze the gene expression data at the level of groups of functionally related genes, such as pathways. Studies have shown that pathway markers tend to be more robust and yield more accurate classification results. One practical problem of the pathway-based approach is the limited coverage of genes by currently known pathways. As a result, potentially important genes that play critical roles in cancer development may be excluded. To overcome this problem, we propose a novel method for identifying reliable subnetwork markers in a human protein-protein interaction (PPI) network.

**Results:**

In this method, we overlay the gene expression data with the PPI network and look for the most discriminative linear paths that consist of discriminative genes that are highly correlated to each other. The overlapping linear paths are then optimally combined into subnetworks that can potentially serve as effective diagnostic markers. We tested our method on two independent large-scale breast cancer datasets and compared the effectiveness and reproducibility of the identified subnetwork markers with gene-based and pathway-based markers. We also compared the proposed method with an existing subnetwork-based method.

**Conclusions:**

The proposed method can efficiently find reliable subnetwork markers that outperform the gene-based and pathway-based markers in terms of discriminative power, reproducibility and classification performance. Subnetwork markers found by our method are highly enriched in common GO terms, and they can more accurately classify breast cancer metastasis compared to markers found by a previous method.

## Background

Given the high-throughput genomic data from microarray experiments, one challenge is to find effective biomarkers associated with a complex disease, such as cancer. Extensive work has been done to identify differentially expressed genes across different phenotypes [1-5], which can be used as diagnostic markers for classifying different disease states or predicting the clinical outcomes [6-11]. However, finding reliable gene markers is very challenging for a number of reasons. The small sample size of typical clinical data is one important factor that makes this problem difficult. We often have to select a small number of gene markers from thousands of genes based on a limited number of samples, which makes the performance of the traditional feature selection methods very unpredictable [12]. In addition to this, the inherent measurement noise in microarray experiments and heterogeneity across samples aggravate this problem further [13-16]. Moreover, previous methods often select gene markers only based on their expression data. Therefore, it is possible that some of the selected gene markers may be functionally related, hence contain redundant information which may lead to the degradation of the overall classification performance.

To address the aforementioned problems, several studies proposed to interpret the expression data at the level of groups of functionally related genes, such as pathways derived from microarray studies [17-19], GO annotations [20], and other sources. Methods have been developed to capture the overall expression changes of a given pathway by jointly analyzing the expression levels of its member genes. For example, Guo et al. [21] used the mean or median expression level of the member genes as the pathway activity. Tomfohr et al. [22] analyzed the expression levels of genes in a pathway using singular value decomposition (SVD), and they used the eigenvector with the largest eigenvalue as the pathway activity. Lee et al. [23] estimated the pathway activity by computing the average expression level of the condition responsive genes (CORGs) within a pathway. More recently, another method has been proposed based on a simple probabilistic model, which estimates the pathway activity that contributes to the phenotype of interest based on the log likelihood ratios (LLR) of the member genes [24]. These pathway-based methods showed that pathway markers are generally more reliable compared to gene markers and that they lead to better or comparable classification performance [21-24]. The main advantage of the pathway-based methods is that they can reduce the effect of the measurement noise and that of the correlations between genes that belongs to the same pathway. Moreover, pathway markers can provide important biological insights into the underlying mechanisms that lead to different disease phenotypes. However, pathway-based methods also have some shortcomings. First, currently known pathways cover only a limited number of genes and may not include key genes with significant expression changes across different phenotypes. Besides, many pathways overlap with each other, hence the activity of such pathway markers may be highly correlated. One possible way to alleviate these problems is to directly identify such markers in a large protein-protein interaction (PPI) network. In a recently published paper [25], Chuang et al. tried to identify subnetwork markers by overlaying gene expression data on the corresponding proteins in a PPI network. They started from the so-called seed proteins in the PPI network that have high discriminative power and greedily grew subnetworks from them to maximize the mutual information between the subnetwork activity score and the class label. They showed that subnetwork markers yield more accurate classification results and have better reproducibility compared to gene markers.

In this paper, we propose a new method for identifying effective subnetwork markers from a PPI network by performing a *global* search for differentially expressed linear paths using dynamic programming. After finding the most discriminative linear paths, we combine overlapping paths into subnetworks through a greedy approach and use those subnetworks as diagnostic markers for classifying breast cancer metastasis. To test the effectiveness of our subnetwork markers, we perform cross validation experiments based on two independent breast cancer datasets. We compare the performance of our method with a gene-based method, a pathway-based method [24] and a previously proposed subnetwork-based method [25]. The results show that the proposed method finds reliable subnetwork markers that can accurately classify breast cancer metastasis. We also perform an enrichment analysis and show that the identified subnetwork markers are highly enriched with proteins that have common GO terms.

## Results and discussion

### Identification of subnetwork markers

We obtained two independent breast cancer datasets from the large-scale expression studies in Wang et al. [10] (referred as the USA dataset) and van’t Veer et al. [9] (referred as the Netherlands dataset). The USA dataset contains 286 samples and the Netherlands dataset contains 295 samples. Metastasis had been detected for 78 patients in the Netherlands dataset and 107 patients in the USA dataset during the five-year follow-up visits after the surgery. The PPI network has been obtained from Chuang et al. [25], which contains 57,235 interactions among 11,203 proteins. Since not all proteins have corresponding genes in the microarray platforms used by the two breast cancer studies, we used the induced network which contains 9,263 proteins and 49,054 interactions for the USA dataset, and 8,380 proteins and 31,201 interactions for the Netherlands dataset.

Our proposed method integrates the gene expression data and the PPI data by overlaying the expression value of each gene on its corresponding protein in the PPI network. The subnetwork identification algorithm consists of the following three major steps:

#### Step 1: Search for highly discriminative linear paths whose member genes are closely correlated to each other

To find discriminative linear paths in the large PPI network, we define a scoring scheme that incorporates both the *t*-test statistics scores of the member genes and the correlation coefficient between their expression values. This scoring scheme takes a weighted sum of the *t*-scores of the member genes within a given path. The weights depend on the correlation between the member genes and the parameter *θ*, where *θ* is introduced to control the trade off between the “discriminative power” of individual genes and the “correlation” between the member genes (see Methods). Based on the above scoring scheme, we developed an algorithm that searches for the top scoring linear paths that have length *l* and end at node *g__i__*.

#### Step 2: Combine top scoring linear paths into a subnetwork

We initialize the subnetwork using the path with the highest score. As long as there exists a high scoring path that overlaps with the current subnetwork, we combine them and check if the discriminative power of the new subnetwork is larger than that of the previous subnetwork. If the discriminative power improves, we keep the new subnetwork. Otherwise, we keep the previous subnetwork and check the next best path. To evaluate the discriminative power of subnetworks, we applied the probabilistic pathway activity inference method proposed in [24] to infer the subnetwork activity. The discriminative power of a subnetwork is assessed by computing the *t*-test statistics score of the subnetwork activity.

#### Step 3: Update the PPI network

After identifying the discriminative subnetwork, we update the PPI network by removing the proteins in the identified subnetwork from the current PPI network. In order to find additional *non-overlapping* subnetworks, we repeat the search process from **Step 1**.

In order to control the size of the identified subnetworks, we restricted the length of the linear paths to be less than 8. For a given *l* and for every node *g_i_* in the network, we identified the top 20 linear paths with the highest scores, whose length is *l* and end at the given node *g_i_*. To construct the subnetwork marker that can be used as a diagnostic marker for breast cancer metastasis, we chose the top 100 scoring linear paths whose length are within a given range 5 ≤ *l* ≤ 8. The selected linear paths were combined into a single subnetwork as described in **Step 2**. To find the best *θ*, we repeated the experiment for six different values *θ* = 1, 2, 4, 8, 16 and ∞. For every value of *θ*, we identified 50 subnetwork markers for each dataset using the proposed method. The statistics of the identified subnetworks for the two datasets are shown in Table 1. We can see that the overlap between the subnetwork markers identified on different datasets is around 25%, which is significantly larger than the overlap reported in Chuang et al. (12.7%) [25].

**Table 1:** Statistics of the subnetwork markers identified by the proposed method.

*θ*			Size	Number of genes	Number of genes in common
				
		mean	standard deviation		
1	USA	16.8	10.17	840	213
	Netherlands	14.62	8.69	731	

2	USA	18.22	12.3	911	233
	Netherlands	16	10.34	801	

4	USA	18	12.8	901	202
	Netherlands	17.28	11.4	864	

8	USA	20.7	13.38	1035	252
	Netherlands	19.52	12.57	976	

16	USA	20.2	11.13	1010	201
	Netherlands	16.64	10.89	832	

∞	USA	22.32	14.86	1116	266
	Netherlands	21.92	10.67	1096	

For each *θ*, we show the mean and standard deviation of the subnetwork size as well as the total number of genes covered by the identified subnetworks. We also show the number of genes shared by the subnetworks identified using the respective breast cancer datasets.

### The identified subnetworks are enriched with proteins that have common GO terms

**Figure 1:**
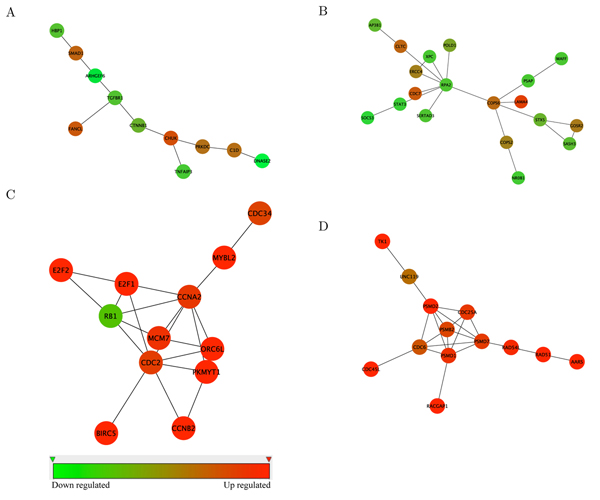
**Sample subnetworks identified using the proposed method.** (A), (B) are examples of subnetworks identified using the USA dataset. (C), (D) are examples of subnetworks identified using the Netherlands dataset. Red (green) implies that the gene is upregulated (downregulated) in breast cancer samples with metastasis.

We identified 50 discriminative subnetworks using the proposed method for both the USA dataset and the Netherlands dataset (*θ* = 8). The identified subnetworks consist of 1035 and 976 genes, respectively. Next, we analyzed the identified subnetworks using FuncAssociate [26], which is a web application designed for characterizing large collections of genes and proteins. It performs a Fisher’s Exact Test (FET) analysis to identify Gene Ontology (GO) [20] attributes that are shared by a fraction of the entries in a given set of genes or proteins. At a significance threshold of 0.01, 78% and 84% of the subnetworks that were respectively identified using the USA dataset and the Netherlands dataset were enriched with proteins that share common GO terms. These GO terms generally correspond to cell growth and death, cell proliferation and replication, cell and tissue remodeling, circulation and coagulation, or metabolism. Examples of the identified subnetworks are shown in Figure 1, where we can see that the proposed method is capable of finding subnetwork markers that also include genes that are oppositely regulated. The enrichment analysis results of the sample subnetworks obtained using FuncAssociate are shown in Table 2.

**Table 2:** Enrichment analysis results for the sample subnetworks shown in Figure 1.

Subnetwork	Attribute ID	*P*-value	Attribute name
A	GO:0045165	0.024	cell fate commitment
	GO:0012501	0.001	programmed cell death
	GO:0008219	0.006	cell death
	GO:0016265	0.006	Death
	GO:0006915	0.017	Apoptosis

B	GO:0000718	< 0.001	nucleotide-excision repair, DNA damage removal
	GO:0006308	0.046	DNA catabolic process
	GO:0043566	0.040	structure-specific DNA binding

C	GO:0051318	0.039	G1 phase
	GO:0022403	< 0.001	cell cycle phase
	GO:0005654	< 0.001	Nucleoplasm
	GO:0000280	0.009	nuclear division
	GO:0007067	0.009	Mitosis
	GO:0048285	0.009	organelle fission
	GO:0051301	0.001	cell division
	GO:0022402	< 0.001	cell cycle process
	GO:0007049	0.000	cell cycle
	GO:0051726	0.008	regulation of cell cycle
	GO:0044428	0.001	nuclear part
	GO:0005634	0.024	Nucleus

D	GO:0005838	< 0.001	proteasome regulatory particle
	GO:0000076	0.016	DNA replication checkpoint
	GO:0032297	0.016	negative regulation of DNA replication initiation
	GO:0030174	0.030	regulation of DNA replication initiation
	GO:0000502	0.010	proteasome complex
	GO:0031145	< 0.001	anaphase-promoting complex-dependent proteasomal ubiquitin-dependentprotein catabolic process
	GO:0051436	< 0.001	negative regulation of ubiquitin-protein ligase activity during mitotic cell cycle
	GO:0051352	< 0.001	negative regulation of ligase activity
	GO:0051437	< 0.001	positive regulation of ubiquitin-protein ligase activity during mitotic cell cycle
	GO:0051444	< 0.001	negative regulation of ubiquitin-protein ligase activity
	GO:0051443	0.001	positive regulation of ubiquitin-protein ligase activity
	GO:0051439	0.001	regulation of ubiquitin-protein ligase activity during mitotic cell cycle
	GO:0051351	0.001	positive regulation of ligase activity
	GO:0051438	0.002	regulation of ubiquitin-protein ligase activity
	GO:0051340	0.002	regulation of ligase activity
	GO:0010498	0.007	proteasomal protein catabolic process
	GO:0043161	0.007	proteasomal ubiquitin-dependent protein catabolic process
	GO:0022402	< 0.001	cell cycle process
	GO:0006511	0.050	ubiquitin-dependent protein catabolic process
	GO:0006259	0.050	DNA metabolic process

### The subnetwork markers identified by the proposed method are more discriminative and reproducible

**Figure 2:**
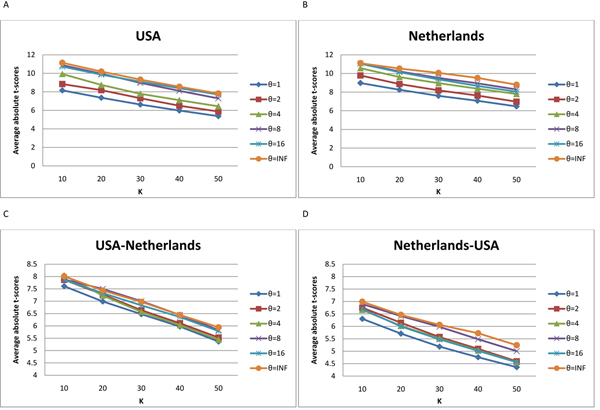
**Discriminative power of the subnetwork markers identified by the proposed method using different *θ*.** We computed the mean absolute *t*-score of the top *K* = 10, 20, 30, 40, 50 subnetwork markers for different values of *θ* (shown in different colors). (A), (B): Markers were identified using a particular dataset and tested on the same dataset. (C), (D): Markers were identified using the first dataset and evaluated on the second dataset.

We first evaluated the subnetwork markers identified using the proposed method. For a given *θ*, we identified the subnetwork markers based on one dataset and estimated their discriminative power on the same dataset. The discriminative power of the subnetwork marker was estimated as the absolute *t*-test statistics score of the subnetwork activity. Subnetwork markers were then sorted in the decreasing order of *t*-score. Next, to show the reproducibility of our subnetwork markers, we identified the top 50 markers based on one dataset and evaluated their discriminative power on the other dataset. Again, subnetwork markers were sorted according to their discriminative power. Figure 2 shows the discriminative power of subnetwork markers identified using six different values of *θ*, where the *x*-axis corresponds to the top *K* markers being considered, and the *y*-axis shows the mean absolute *t*-score of the top *K* markers (*K* = 10, 20, 30, 40, 50). Figure 2A and Figure 2B show the results obtained from the USA dataset and the Netherlands dataset, respectively. Figure 2C shows the discriminative power of the subnetwork markers selected based on the Netherlands dataset and evaluated using the USA dataset. Figure 2D shows the discriminative power of the markers selected based on the USA dataset and evaluated using the Netherlands dataset. As we can see from these results, the discriminative power of the identified subnetwork markers is not very sensitive to the choice of *θ*. To further compare the identified subnetwork markers with other markers, we used *θ* = 8 which showed good performance in average.

Next, we compared the identified subnetwork markers with gene markers, pathways markers [24] and the subnetwork markers identified by Chuang et al. [25]. For gene markers, we selected the top 50 genes based on the absolute *t*-score among all genes covered by the 50 identified subnetworks. For pathway markers, we selected the top 50 pathways among the 639 pathways in the C2 curated gene sets in MsigDB (Molecular Signatures Database) [17]. We also obtained the subnetworks identified by Chuang et al. [25] from the Cell Circuits database [27] (149 discriminative subnetworks for the Netherlands dataset and 243 subnetworks for the USA dataset). We chose the top 50 subnetworks out of 149 subnetworks based on the Netherlands dataset and the top 50 subnetworks out of 243 subnetworks based on the USA dataset. The pathways and subnetworks were ranked using the scheme proposed by Tian et al. [18], based on the average absolute *t*-test statistics score of all the member genes. For subnetwork markers identified by Chuang et al., we computed the *t*-scores of their member genes using the original expression values. For pathway markers, *t*-scores of the member genes were computed using their log-likelihood ratios as in [24] (see Methods). To assess the discriminative power of the subnetwork markers identified using the proposed method, their activity score was inferred using the probabilistic inference method proposed in [24]. For subnetwork markers identified by Chuang et al., we inferred their activity score using the mean expression value of the member genes as reported in their paper [25].

**Figure 3:**
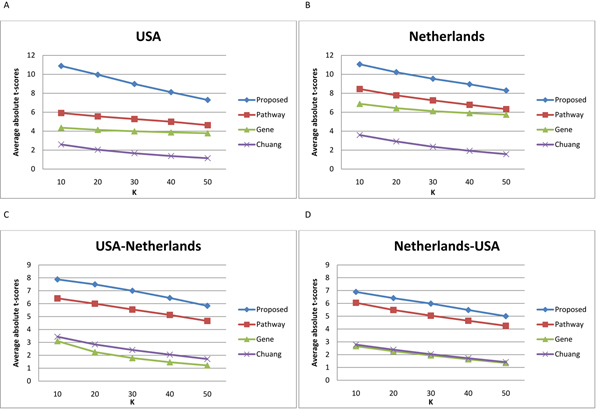
**Discriminative power of different types of markers.** We evaluated the discriminative power of the subnetwork markers identified using the proposed method, and compared them with gene markers, pathway markers [24], and the subnetwork markers identified by Chuang et al. [25]. Mean absolute t-score is shown for the top *K* = 10, 20, 30, 40, 50 markers. (A), (B): Markers were identified using a particular dataset and tested on the same dataset. (C), (D): Markers were identified using the first dataset and evaluated based on the second dataset.

The discriminative power of these different markers are shown in Figure 3. As we can see in Figure 3, subnetwork markers identified by our method are more discriminative compared to other markers. Moreover, it can be seen that they also retain higher discriminative power across different datasets.

### Subnetwork markers identified by the proposed method improve classification performance

To evaluate the performance of the classifiers that are constructed using the subnetwork markers identified by the proposed method, we performed the following within-dataset and cross-dataset cross-validation experiments.

In the within-dataset experiments, the top 50 subnetwork markers identified using one of the two breast cancer datasets were used to build the classifier. The dataset was divided into ten folds of equal size, one of them was withheld as the “test set” and the remaining nine were used for training the classifier. In the training set, six folds (referred as the “marker ranking set”) were used to rank the subnetwork markers according to their discriminative power and to build the classifier using logistic regression. The other three folds (referred as the “feature selection set”) were used for feature selection. We started with the top ranked subnetwork marker and enlarged the feature set by adding features sequentially. Every time we included a new subnetwork marker into the feature set, a new classifier was built using the marker ranking set and it was tested on the feature selection set. For all the samples in the feature selection set, the classifier can compute the posterior probabilities of the class label (metastasis versus metastasis-free), based on which we can estimate the AUC (Area Under ROC Curve) [28]. The AUC metric provides a useful statistical summary of the classification performance over the entire range of sensitivity and specificity. We retained the new subnetwork marker if the AUC (estimated on the feature selection set) increased; otherwise, we discarded the subnetwork marker and continued to test the remaining ones. The above experiment was repeated 500 times based on 50 random ten-fold splits. The average AUC was reported as the classification performance measure.

To evaluate the reproducibility of the subnetwork markers, we performed the following cross-dataset experiments. We first identified the top 50 subnetwork markers based on one dataset and performed cross-validation experiments on the other dataset, following a similar procedure that was used in the previously described within-dataset experiments.

**Figure 4:**
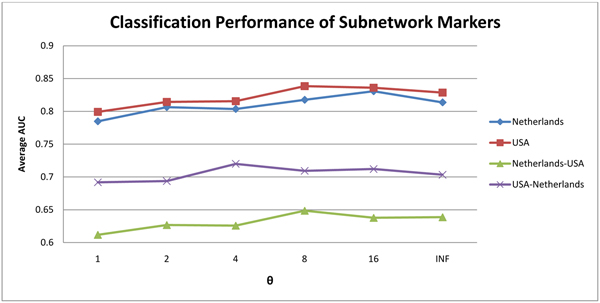
**Classification performance of the identified subnetwork markers for different *θ*.** The line plots show the average AUC for classifiers based on subnetwork markers identified using *θ* = 1, 2, 4, 8, 16, ∞. The legends USA, Netherlands denote the results of within-dataset experiments based on the USA dataset and the Netherlands dataset, respectively. The legends USA-Netherlands, Netherlands-USA denote the results of cross-dataset experiments where markers were identified based on the first dataset and tested based on the second dataset.

For comparison, we also performed similar within-dataset and cross-dataset experiments using gene markers, pathway markers and the subnetwork markers identified by Chuang et al., respectively. For each method, we limited the feature set to the top 50 markers for each dataset. Figure 4 shows the classification performance based on the subnetwork markers identified by the proposed method for different values of *θ*. We found that the AUC for both within-dataset and cross-dataset experiments first increases with increasing *θ* and starts to drop after certain point. At *θ* = 8, the AUC values for both cross-dataset experiments are relatively larger than those at other values of *θ*. Also, the AUC values for both within-dataset experiments at *θ* = 8 compare favorably with those at different *θ*, which implies that the trade off between maximizing the discriminative power and increasing the correlations of the member genes is well balanced.

**Figure 5:**
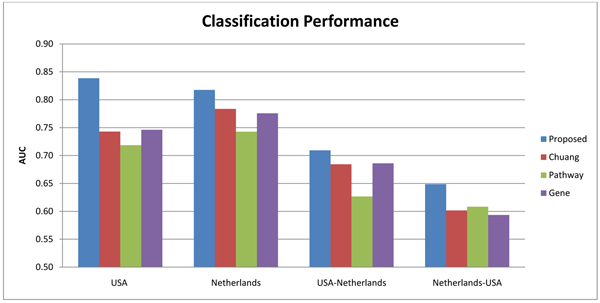
**Classification performance of different types of markers.** The bar charts show the average AUC of different classifiers that use subnetwork markers identified by the proposed method, gene markers, pathway markers, and subnetwork markers found by Chuang et al.’s method. Results of the within-dataset experiments based on the USA and Netherlands dataset are shown in the two bar charts on the left. The two bar charts on the right show the results of the cross-dataset experiments, where markers were identified based on the first dataset and tested based on the second dataset.

To compare the classification performance of the identified subnetwork markers with other types of markers, we set *θ* = 8. Based on this setting, we compared our subnetwork markers with gene markers, pathway markers and the subnetwork markers from Chuang et al. using the experimental designs described above. Figure 5 summarizes the classification performance of the proposed approach, in comparison with the other methods. The two bar charts on the left of Figure 5 show the AUC of the within-dataset experiments. As shown in Figure 5, classifiers based on the subnetwork markers identified by the proposed method perform significantly better than the classifiers based on other types of markers. The results of the cross-dataset experiments are shown in the two bar charts on the right of Figure 5. Again, we can see that the classifiers built on the subnetwork markers predicted by our method significantly outperform those based on other markers. This indicates that the predicted subnetwork markers are more reproducible compared to other markers.

**Figure 6:**
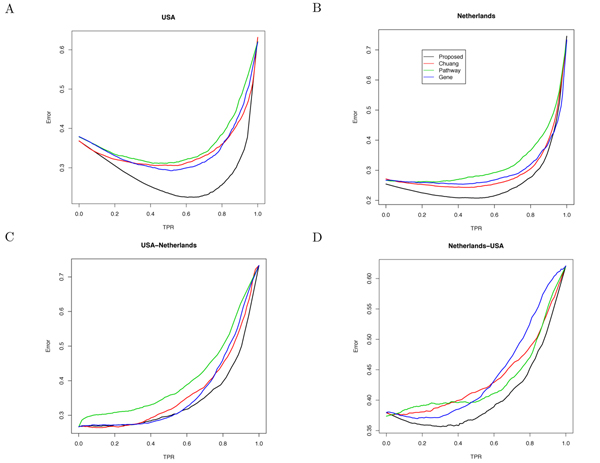
**Classification error at different TPR (true positive rate) for different types of markers.** (A), (B) show the results of the within-dataset experiments based on the USA dataset and the Netherlands dataset, respectively. (C), (D) show the results of the cross-dataset experiments, where markers were identified using the first dataset and tested based on the second dataset.

Figure 6 shows the classification error of the classifiers built using different types of markers at different TPR (true positive rate). As shown in Figure 6, the error curve that corresponds to the proposed markers always lies below others, which implies that classifiers built on our subnetwork markers yield a lower error rate at any fixed sensitivity level.

## Conclusions

In this paper, we proposed a new method for identifying effective subnetwork markers in a protein-protein interaction (PPI) network. As shown throughout this paper, integrating the PPI network with microarray data can overcome some of the shortcomings of the gene-based and pathway-based methods. First of all, using a genome-scale PPI network provides a better coverage of the genes in the microarray studies compared to using known pathways obtained from public databases. Second, the network topology provides prior information about the relationship between proteins, hence the genes that code for these proteins. Subnetworks identified by integrating the network structure and the gene expression data can cluster proteins (or genes) that are functionally related to each other. By aggregating the expression values of the member genes, subnetwork markers can avoid selecting single gene markers with redundant information. Furthermore, the discriminative subnetworks identified by the proposed method can also provide us with important clues about the biological mechanisms that lead to different disease phenotypes. The proposed method finds top scoring linear paths using dynamic programming and combines them into a subnetwork by greedily optimizing the discriminative power of the resulting subnetwork marker. We developed a scoring scheme that is used by the search algorithm to find linear paths that consist of discriminative genes that are highly correlated to each other. The proposed algorithm allows us to control the trade off between maximizing the discriminative power of the member genes within a given linear path and increasing the correlation between the member genes, by choosing the appropriate value for *θ*. As the subnetwork markers are constructed based on the top scoring linear paths, instead of single genes, the proposed method is expected to yield more robust subnetwork markers. Another important advantage of our method is that it can find non-overlapping subnetwork markers. This can reduce the overall redundancy among the identified markers. In this paper, the activity of the identified subnetwork markers were inferred using the probabilistic activity inference scheme proposed in [24]. This allows us to find better subnetwork markers, since it can assess their discriminative power more effectively.

As shown in this paper, the identified subnetwork markers consist of proteins that share common GO terms. The classifiers based on the subnetwork markers identified using the proposed method were shown to achieve higher classification accuracy in both within-dataset and cross-dataset experiments compared to classifiers based on other markers. These results suggest that the method proposed in this paper can find effective subnetwork markers that can more accurately classify breast cancer metastasis and are more reproducible across independent datasets.

## Methods

### Overview

**Figure 7:**
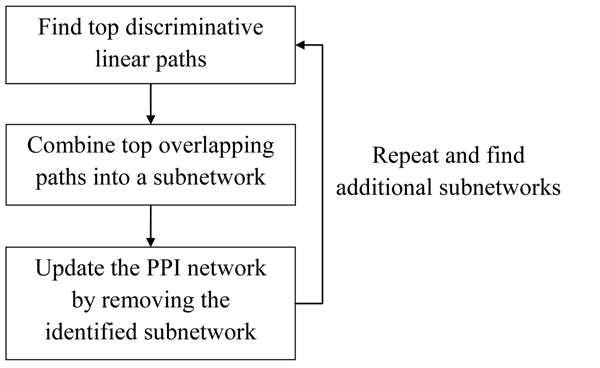
Illustration of the proposed method.

Given a large PPI network, we want to find subnetwork markers whose activity is highly indicative of the disease state of interest. For this purpose, we first need a method for inferring the activity of a given subnetwork and evaluating its discriminative power. There exist different ways for computing the activity score of a given group of genes [24]. Recently, we proposed a probabilistic pathway activity inference scheme, which was shown to outperform many other existing methods. Thus, we adopt this activity inference scheme for finding subnetwork markers whose activity scores are highly discriminative of the disease states. However, finding the subnetwork markers with maximum discriminative power in a PPI network based on the selected inference method is computationally infeasible. For this reason, we propose an algorithm for identifying effective subnetwork markers which is motivated by a simple scheme proposed in Tian et al. [18]. This scheme scores a pathway marker by computing the average absolute *t*-score of its member genes. It has been shown to be effective in evaluating the discriminative power of pathway markers in [24]. Since our goal is to find groups of genes that display coordinated expression patterns, we modified Tian et al.’s scoring scheme to incorporate the correlation between the genes within a given pathway. This new method scores a given pathway by taking the weighted sum of the absolute *t*-scores of its member genes, where the weights are computed using the correlation coefficients between the member genes. The general outline of the proposed algorithm is as follows. Based on the above scoring scheme, we first search for differentially expressed linear paths in the PPI network. Then, the top paths that overlap with each other are greedily combined into a subnetwork by maximizing the discriminative power of the resulting subnetwork, evaluated by the method proposed in [24]. The identified subnetwork is removed from the PPI network, and the above process is repeated to find multiple non-overlapping subnetwork markers. The overall scheme is illustrated in Fig. 7.

### Probabilistic inference of subnetwork activity

Here we provide a brief review of the probabilistic activity inference method proposed in [24]. Suppose we have a subnetwork *G_s_* that consists of *n* proteins which correspond to *n* different genes {*g*_1_, *g*_2_,…,*g_n_*}. Assume that the expression level *x_i_* of a gene *g_i_* follows the distribution  under phenotype *k* = 1, 2. The log-likelihood ratio (LLR) [24] between the two phenotypes is computed as follows

In order to estimate the conditional probability density function , we assume that the gene expression level of gene *g_i_* under phenotype *k* follows a Gaussian distribution with mean  and standard deviation . The parameters are empirically estimated using the samples with phenotype *k*. Given the log-likelihood ratio of each gene, the subnetwork activity  is defined as the sum of the log-likelihood ratios of the member genes . 

### Evaluating the discriminative power of linear paths in the PPI network

A linear path λ = {*g*_1_, *g*_2_,…,*g_n_*} in a given PPI network *G* is defined as a group of genes, where the proteins that correspond to *g_i_* and *g_i+_*_1_ are connected for *i* = 1,…, *n −* 1. To evaluate the discriminative power of a linear path, we first evaluate the discriminative power of each gene *g_i_* by computing the *t*-test statistics score of the log-likelihood ratio *α*(*x_i_*), denoted as *t_α_*(*g_i_*). Then, we compute the Pearson product-moment correlation coefficient to measure the correlation between the log-likelihood ratios of ∀*g_i_*, *g_j_* ∊ λ. The correlation matrix is given by

where *ρ_ij_, i* ≠ *j* is the correlation coefficient between the log-likelihood ratios of *g_i_* and *g_j_*. The score of the pathway λ is defined as following

where  (*I* is the identity matrix), and *J* is an all-one-column vector. We use a normalization factor of  to ensure that the overall score does not depend on the length of the path. We use *θ* to control the trade off between maximizing the discriminative power of the genes within the identified path and increasing the correlation between its member genes. When *θ* = 0, the weight for the *t*-score of a given gene *g_i_* is determined by the average correlation between the log-likelihood ratios of *g_i_* and *g_j_*, where *j* ≠ *i*. As *θ* increases, we give more weight on the discriminative power of individual genes than the correlation between member genes. Especially, when *θ* → ∞, we get Σ′(λ) = *I*. In this case, the pathway score *S*(λ) is simply the average *t*-score of the member genes in λ, and the proposed subnetwork marker identification method reduces to its preliminary version proposed in [29]. The above scoring scheme is used for finding the top linear paths in the network *G* as we describe in the following section.

### Searching for discriminative linear paths

Let *G* = (*E*, *V*) denote the PPI network, where *V* is the set of nodes (i.e., proteins), *E* is the set of edges (i.e., protein interactions). Suppose there are *N* proteins in *G*. Then we can represent *E* as an *N*-dimensional binary matrix. For any protein pair (*v_a_*, *v_b_*), where *v_a_*, *v_b_* ∊ *V*, we let *E*[*v_a_*, *v_b_*] = 1, if *v_a_*, *v_b_* are connected; *E*[*v_a_*, *v_b_*] = 0, otherwise. Based on the scoring scheme defined in the previous section, we search for top discriminative linear paths using dynamic programming. We define λ(*v_i_*, *l*) as the optimal linear path among all linear paths that have length *l* and end at *v_i_*. The score of this optimal path is defined as

*s*(*v_i_, l*) = *t_α_*[λ(*v_i_,l*)] Σ′[λ(*v_į_*, *l*)].

Here, only paths with length *l* ≤ *L* are considered. The algorithm is defined as follows.

(i) **Initialization:***l* = 1, ∀*v_i_*∊ *V*,

*s*(*v_i_, l*) = |*t_α_*(*v_i_*)|.

(ii) **Iteration:**

for *l* = 2 to *L*,

for ∀*v_i_*∊ *V*,

if *s*(*v_i_*, *l*) > 0, then

end

end

(iii)** Termination:**

for ∀*v_i_*∊ *V*, 1 ≤ *l* ≤ *L*,

*S*(λ(*v_i_*, *l*)) = *s*(*v_i_*, *l*)/*l*^2^.	(1)

Although the above algorithm finds only the top path for every (*v_i_*, *l*), we can easily modify it to find the top *M* discriminative paths. Increasing *M* allows us to find better linear paths with higher discriminative power, but it will also increase the computational complexity of the algorithm.

### Combining top overlapping paths into a subnetwork

Based on (1), we choose the *m* top scoring paths Λ = {λ_1_, λ_2_, … , λ*_m_*} whose length is within a given range [*L*_min_, *L*_max_]. Next, the paths in Λ are combined into a subnetwork *G_s_* so that its discriminative power *R*(*G_s_*) is locally optimized. This process is carried out as follows:

(i) *G_s_* ← λ*_i_*, *G_temp_* ← *G_s_*, *i* = 1.

(ii) *i* =* i* + 1; If λ*_i_* ∩ *G_s_* ≠ ø,* G_temp_* ← *G_temp_* ∪ λ*_i_*.

(iii) If *R*(*G_temp_*) > (1 +*∊*)*R*(*G_s_*), *G_s_ ← G_temp_*; else* G_temp_ ← G_s_*.

(iv) Go to (ii) if *i < m;* otherwise, terminate.

Here *∊* is set as 0.01 to avoid over-fitting to the expression data. We used the activity inference method in [24] to computed the actual activity score of *G_s_*. Then, *R*(*G_s_*) is computed as the *t*-test statistics of the subnetwork activity score.

After obtaining a subnetwork *G_s_*, we removed it from the network *G* by setting *E*[*v_s_*, *v_i_*] = *E*[*v_i_*, *v_s_*] = 0, ∀*v_s_εG_s_*,*v_i_*∊ *G.* Then, the whole process was repeated using the updated network to find additional subnetwork markers.

## Authors contributions

Conceived and designed the experiments: JS BJY ERD. Performed the experiments: JS. Analyzed the data: JS BJY ERD. Wrote the paper: JS BJY ERD.

## Competing interests

The authors declare that they have no competing interests.
